# The effect of nutritional biochemical indexes on the hospitalization outcome of COVID-19

**DOI:** 10.18632/aging.205335

**Published:** 2023-12-13

**Authors:** Peng Wang, Wenying Shi, Xiaodi Zhao, Guanan Zhao, Lidan Ding, Sen Zhang, Jiaxin Li

**Affiliations:** 1 Department of Clinical Laboratory, The Sixth Affiliated Hospital of Wenzhou Medical University and Lishui City People’s Hospital, Lishui 323000, Zhejiang Province, China; 2 State Key Laboratory of Bioactive Substances and Functions of Natural Medicines, Institute of Materia Medica, Chinese Academy of Medical Sciences and Peking Union Medical College, Beijing 100050, China; 3 State Key Laboratory of Functions and Applications of Medicinal Plants, College of Pharmacy, Guizhou Provincial Engineering Technology Research Center for Chemical Drug R&D, Guizhou Medical University, Guiyang 550004, Guizhou, P.R. China; 4 Department of Urology Surgery, The Sixth Affiliated Hospital of Wenzhou Medical University and Lishui City People’s Hospital, Lishui 323000, Zhejiang Province, China; 5 Department of Clinical Nutrition, The Sixth Affiliated Hospital of Wenzhou Medical University and Lishui City People’s Hospital, Lishui 323000, Zhejiang Province, China

**Keywords:** cytokines, COVID-19, PBMC, nutritional biochemical indicators, hospitalization outcomes

## Abstract

Aims to investigate the relationship between nutritional biochemical indexes and hospitalization outcomes of COVID-19 patients, 132 continuous patients with COVID-19 from December 2022 to January 2023 in Lishui hospital were retrospectively analyzed, and the nutritional biochemical indexes in peripheral blood, such as total protein, albumin, calcium, phosphorus, and magnesium, were detected. Meanwhile, the levels of several cytokines and PBMC subtypes (CD4, CD3, CD8, NK and B cells) were detected too. The Spearman correlation analysis, one-way ANOVA and multivariate logit regression were conducted. Results suggested that the levels of total protein and albumin were significantly decreased in patients with poor outcomes, and the levels of calcium, phosphorus, and magnesium were significantly correlated with hospitalization outcomes. COVID-19 patients with diabetes had higher levels of IL-6 and IFN-γ than those patients without diabetes. The levels of IL-2, IFN-γ, IL-6 and Il-10 in the dead patients were significantly higher than those in the recovery and worse patients. Total protein and albumin were significantly positively correlated with levels of NK and B, CD4, CD8, CD3 lymphocytes. The levels of CD4, CD8 and CD3 lymphocytes were significantly decreased in dead patients than other patients. Multivariate logit regression analysis suggests that lymphocyte number, albumin and IL-6 are independent risk factors to evaluate the hospitalization outcome. In summary, nutritional biochemical indexes were significantly corelated with cytokines and PBMC subsets, and had an impact on the severity of COVID-19 patients. Improvement of low protein malnutrition is broad-spectrum and basic strategy to improve the hospitalization outcome of COVID-19.

## INTRODUCTION

Coronavirus disease 2019 (COVID-19) began in late 2019 in Wuhan, China [1], and spread worldwide, causing a pandemic and a global shutdown around the world. Due to China’s complete liberalization of epidemic prevention and control of omicron in December 2022, many medical institutions are encountering large numbers of patients infected with COVID-19, and all the subtypes belonged to BA.5.2 or BF.7 [2]. We know that nutrition-related factors such as high protein levels and nutritional support can reduce the susceptibility to COVID-19 [3] and the secretion of cytokines after COVID-19 infection, improve multiple organ dysfunction syndrome (MODS) [4] and death caused by cytokine outbreak [5]. It is also concerned with increased secretion of cytokines and mortality in COVID-19 patients with diabetes mellitus. However, the effect of nutritional biochemical markers such as calcium [6], phosphorus and magnesium [7] on the prognosis of COVID-19 has not been clarified. The aim of this study was to analyze the influence of nutritional biochemical indexes on the hospitalized outcomes of patients with COVID-19 based on cytokine secretion and PBMC subgroup expression from the nutritional perspective [8]. Cytokines are important inflammation mediators, and under certain pathological conditions, cytokines may act on distal cells in an endocrine manner, thereby mediating systemic reactions. Elevated Proinflammatory cytokines are characteristic of the inflammatory cytokine storm syndrome (CSS) [9]. Pathogenic microbes cause excessive proliferation and activation of T cells and macrophages, leading to dysregulation of cellular immunity [10, 11]. In addition, combined with dendritic cells (DCS), natural killer (NK) cells also activated by COVID-19, leading to cytokine storms that destroy target cells [12]. The overall evaluation of nutritional biochemical indexes in hospitalized outcomes analysis of COVID-19 based on cytokine and mononuclear cells analysis, as well as its value as a guideline for nutritional therapy has been reported. In the current study, with the help of clinical resources in our hospital, we are trying to continue to further clarify the relationship between patients’ nutrient status with their corresponding immunity, and highlight the effects of nutrients supplements on the immune system and their possible benefits in combating the harms caused by infection with the COVID-19 virus [13]. At last, univariate and multivariate ordered logistic regression were performed to evaluate which nutrient related factors were important for COVID patient outcome.

## MATERIALS AND METHODS

### Clinical data

The current study was a retrospective observational study conducted at Lishui City People’s Hospital. Total of 132 consecutive patients were admitted to Lishui City People’s Hospital with COVID-19 (BA.5.2 or BF.7) from December 20^th^, 2022, to January 20^th^, 2023. Patients with incomplete or unavailable clinical data, or with a previous diagnosis of human immunodeficiency virus (HIV), endocrine disorders, or autoimmune disease, blood lymphoid carcinoma were excluded from the study.

This data was divided into two batches, the first with 98 specimens were collected from December-26^th^, 2022 to December-29^th^, 2022, and the second with 34 samples were collected from 10^th^-January, 2023 to 18^th^- January, 2023. Because the infection time was basically from 22^th^- December, 2022 to 29^th^-December, 2022 in Lishui City, and the cytokines decreased gradually with time, so the cytokines levels of second batch were significantly lower than the first batch. Therefore, in the following cytokine correlation study, only the 98 samples collected from the first batch were used for statistical analysis.

### Laboratory parameters

Nutrition-related biochemical indicators (peripheral blood albumin, total protein, calcium, magnesium, and phosphorus) were examined by automated biochemical analyzer (Accute, TBA - 40 FR, Tokyo, Japan), Cytokine-related indicators (IL-2, IL-4, IL-6, IL-10, IL-17a, IFN-γ, and TNF-α) were measured by MILLIPLEX® Multiplex Assays Using Luminex® Technology (Merck, Shanghai, China); PBMC subgroup (CD3, CD3%, CD4, CD4%, CD8, CD8%, CD4/CD8, NK, NK%, B, B%) were detected by FACSCanto II flow cytometer (BD Biosciences, Franklin Lakes, NJ, USA) [14]. All the antibodies of immune cell biomarkers which were suitable for flow cytometry were purchased from BioLegend Ltd (San Diego, CA, USA).

We collected all demographic, clinical, laboratory, and immune parameters at hospital admission. We followed up with patients until hospital discharge or death. Then, we formed three groups of patients according to the primary outcome: 1. recovery group; 2. poor or worse group; 3. death group. All the demographic information about patients was shown in Table 1.

**Table 1:** Clinical parameters in each group.

**Parameters**	**Total (N=132)**	**Recovery (N)**	**Poor or worse (N)**	**Death (N)**
Gender				
men	70	36	17	17
women	62	41	10	11
Age (years)	67.5±17.1	64.4±17.6	74.3±13.2	70.3±16.3
With diabetes	32	15	7	10
No diabetes	100	62	20	18
ventilator use	41	2	17	22
Non ventilator use	91	75	10	6
severity				
Mild to moderate	60	50	7	3
Severe	72	27	20	25

### Statistical methods

SPSS 23.0 (SPSS, Chicago, IL, USA) and GraphPad Prism 8.0 (GraphPad Prism, San Diego, USA) were used for data entry, statistics, and analysis. Descriptive statistical analysis and Shapiro-Wilk method were used to test normality distribution of the measurement data. Chi-square and unpaired Student’s *t*-tests were used for comparison between two groups, and one-way ANOVA post-hoc tests with the Bonferroni correction was conducted for multiple group comparison. Data was shown as mean ± standard deviation. Pearson chi-square test was used for nonparametric test. Univariate and multivariate ordered logistic regression analysis was used to screen for independent risk factors significantly associated with clinical outcomes, which was conducted by SPSS. *P*-value less than 0.05 was considered as significant [15].

### Availability of data and materials

All the data are available on requirement.

### Patient consent for publication

All patients consented for publication.

## RESULTS

### The total protein and albumin in plasma are correlated with hospitalization outcomes

**Figure 1:**
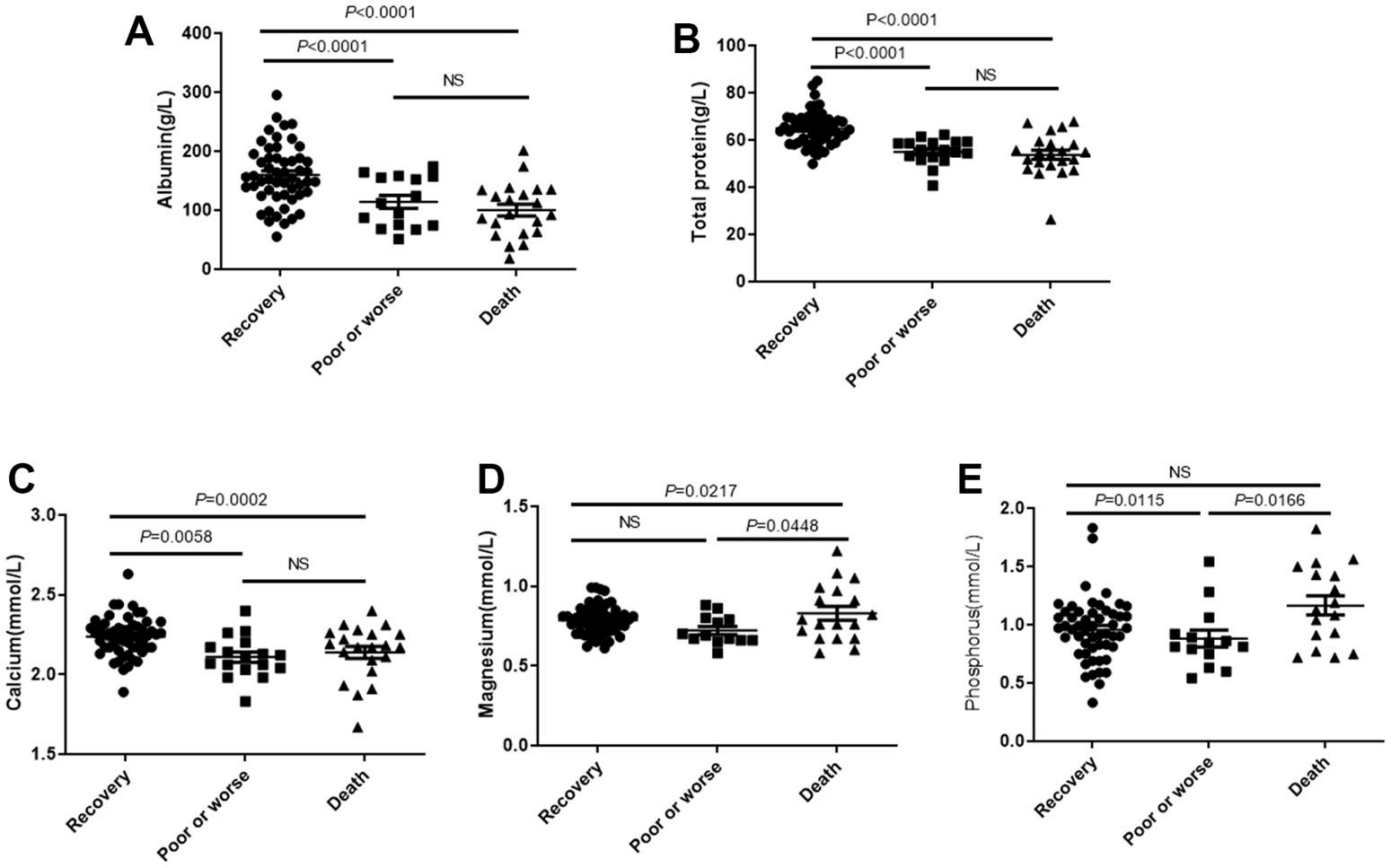
**The plasma total protein and albumin levels affects outcomes of patients with COVID-19.** Patients were divided into the recovery group, poor or worse group and the death group. (**A**, **B**) The total protein and albumin levels in 3 groups. (**C**–**E**) The distribution of concentration of calcium, phosphorus and magnesium in three groups. NS: not significant.

There were significant differences in total protein (Figure 1A) and albumin levels (Figure 1B) among the recovery group, poor or worse group and the death group, and the hospitalization outcomes of the patients with low albumin and total protein was significantly worse than those with high albumin and total protein. The concentration of calcium in recovery group was significantly higher than in worse or death group, but no statistical difference between worse and death group (Figure 1C). Magnesium concentration is contrary to Calcium, and had highest concentration in death group, compared with worse or recovery group (*P*<0.05), but no statistical difference between recovery group and worse group (Figure 1D). Phosphorus concentration in plasma is abnormal in worse group, its plasma concentration is significantly lower than recovery or death group, both *P*<0.05, but no statistical difference was found between recovery and death group (Figure 1E).

The mortality was higher in patients with diabetes (31%) than those patients without diabetes (18%), however, there was not statistically significance between the two groups (Table 2).

**Table 2:** Clinical outcome distribution based on diabetes.

**Grouping**	**Death**	**Survival**	**P-value^#^**
With diabetes	10 (31%)	22 (69%)	0.0914
Without diabetes	18 (18%)	82 (82%)	

#, by Fisher’s exact test.

### Total protein and albumin levels are correlated with peripheral inflammatory cytokines

**Figure 2:**
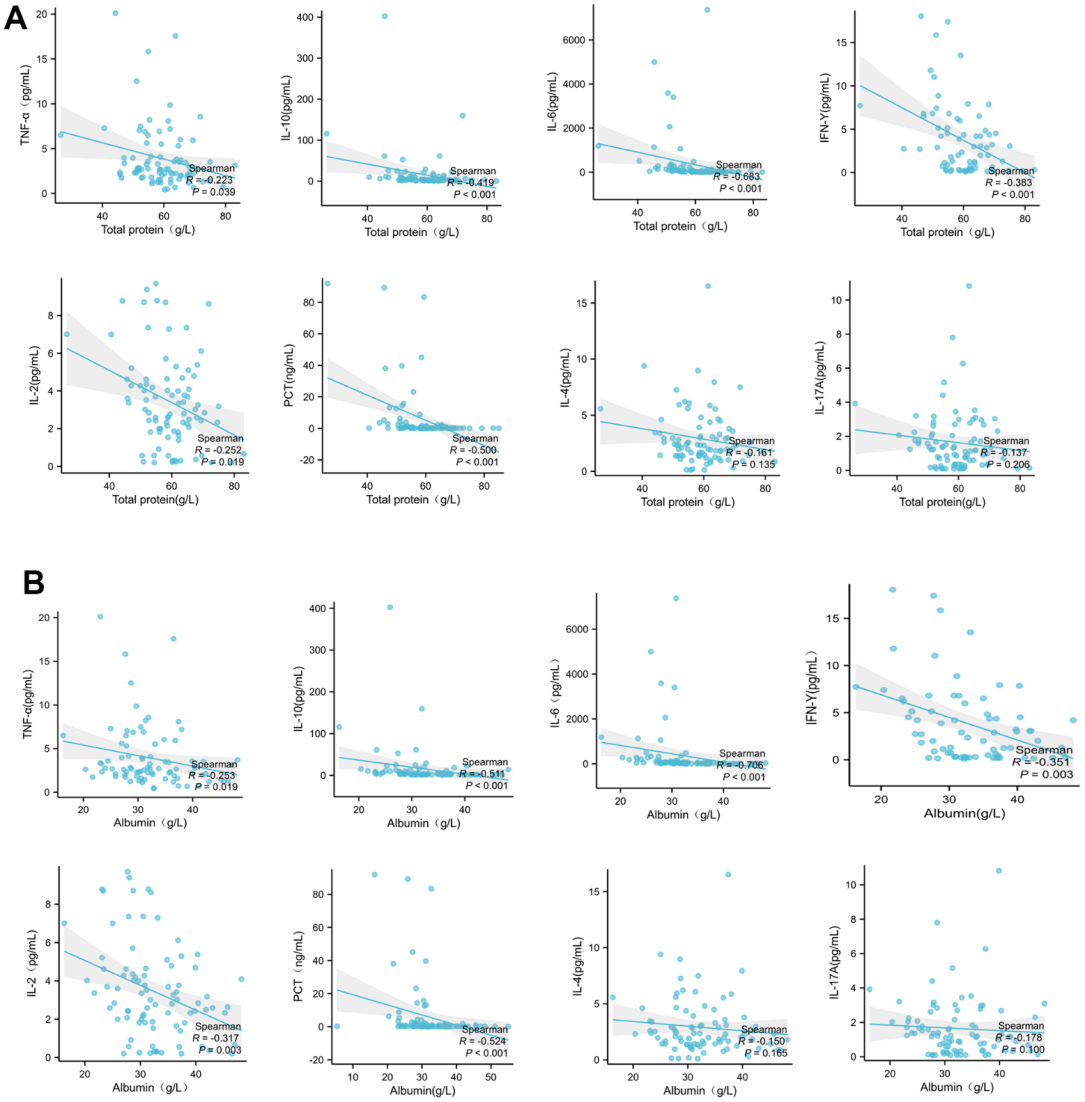
Plasma total protein (**A**) or albumin (**B**) levels in patients with COVID-19 are correlated with peripheral inflammatory cytokines. The correlation between total protein with peripheral inflammatory factors were analyzed by Spearman correlation analysis.

The correlation between plasma total protein and albumin with peripheral inflammatory factors were analyzed by Spearman correlation analysis. As showed in Figure 2A, the total protein levels were negatively associated with peripheral levels of TNF-α, IL-10, IL-6, IFN-γ, IL-2 and procalcitonin (PCT) significantly, but not with IL-4 and IL-17a. In agreement with plasma total protein, the level of albumin was also correlated with above peripheral inflammatory cytokines as same trend as total protein with statistical difference (Figure 2B).

### Death and diabetes contribute to the alteration of cytokines

**Figure 3:**
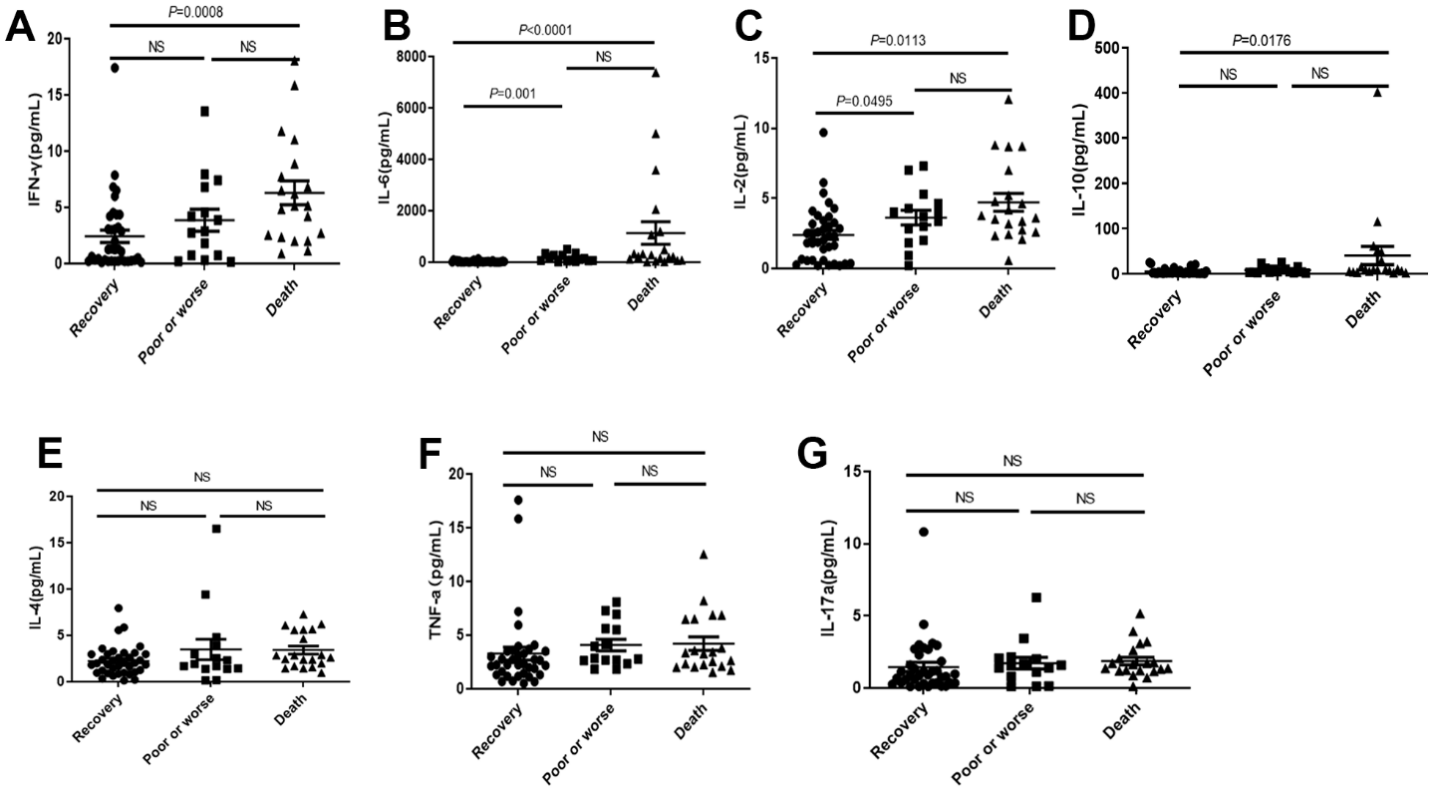
**Distribution of cytokines in the dead, poor or worse and recovery groups with COVID-19.** (**A**–**D**) The IL-2, IFN-γ, IL-6 and IL-10 levels were significantly higher in death group than those in recovery, and poor or worse group. (**E**–**G**) No significant difference in IL-4, TNF-α and IL-17a among three groups. NS: not significant.

IL-2, IFNγ, IL-6 and IL-10 were significantly higher in death group than those in recovery group and poor or worse group (Figure 3A–3D), but no significant difference on IL-4, TNF and IL-17a among three groups (Figure 3E–3G).

The mortality (31%) was higher than that in patients with diabetes than those patients without diabetes (18%), but it was not statistically significant as Table 2.

**Figure 4:**
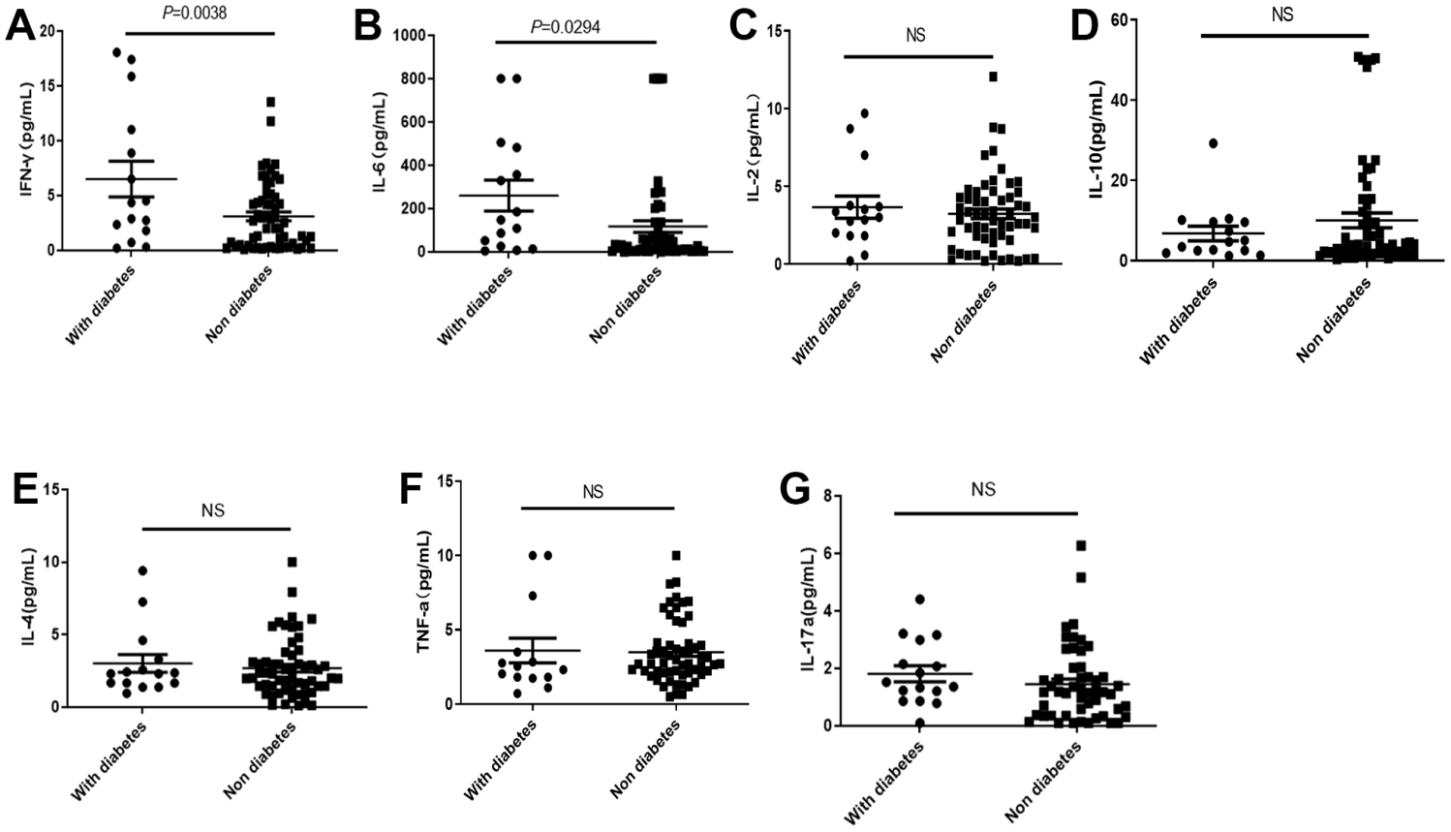
**Distribution of cytokines in the COVID-19 patients with or without diabetes.** (**A**, **B**) The IL-2 and IL-6 in COVID-19 patients with diabetes were significantly higher than those without diabetes. (**C**–**G**) There was no significant difference in IL2, IL-10, IL-4, TNF-α, and IL-17a levels in COVID-19 patients with or without diabetes. NS: not significant.

Although diabetes did not cause significant rise of mortality, the IFNγ and IL-6 in COVID-19 patients with diabetes were significantly higher than those without diabetes (Figure 4A, 4B), while there were no significant differences in IL-2, IL-4, IL-10, TNF-α and IL-17a levels dependent with diabetes (Figure 4C–4G).

### Plasma total protein and albumin levels are correlated with PBMC subsets

**Figure 5:**
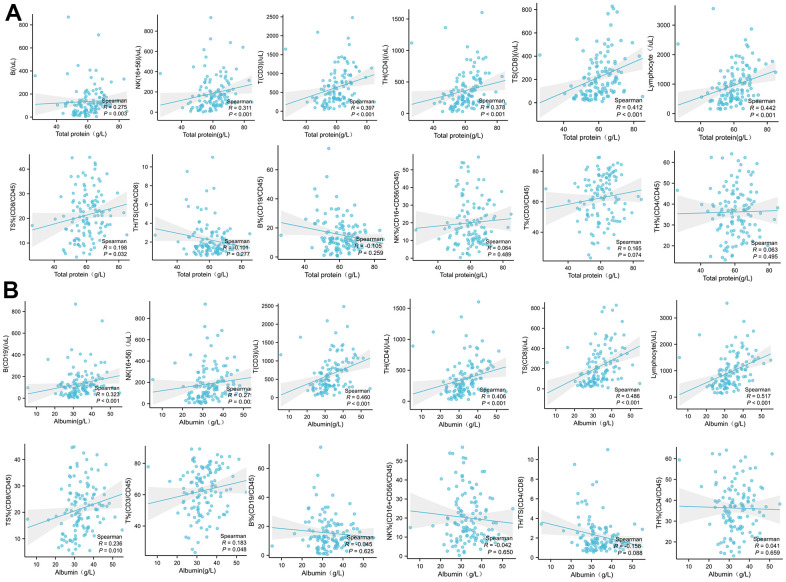
The correlation analysis between the plasma total protein (**A**) or albumin (**B**) levels in COVID-19 patients with the absolute concentration or frequency (%) of B, NK, CD3, CD4, CD8, and total lymphocytes in PBMC.

The total protein level in peripheral blood was significantly and positively correlated with the absolute concentration of B, NK, CD3, CD4, CD8, and total lymphocytes, and also with the frequency of CD8 cells, while with no effect on the frequency of other immune cells (Figure 5A).

The peripheral albumin levels were significantly positively correlated with the absolute concentration of B, NK and CD3, CD4, CD8, and total lymphocytes, and furthermore with the frequency of CD8 and CD3 lymphocytes. However, there was no significant correlation with the frequency of B, NK, and CD4 (Figure 5B).

### The concentration of peripheral T lymphocytes decreases in death cases

**Figure 6:**
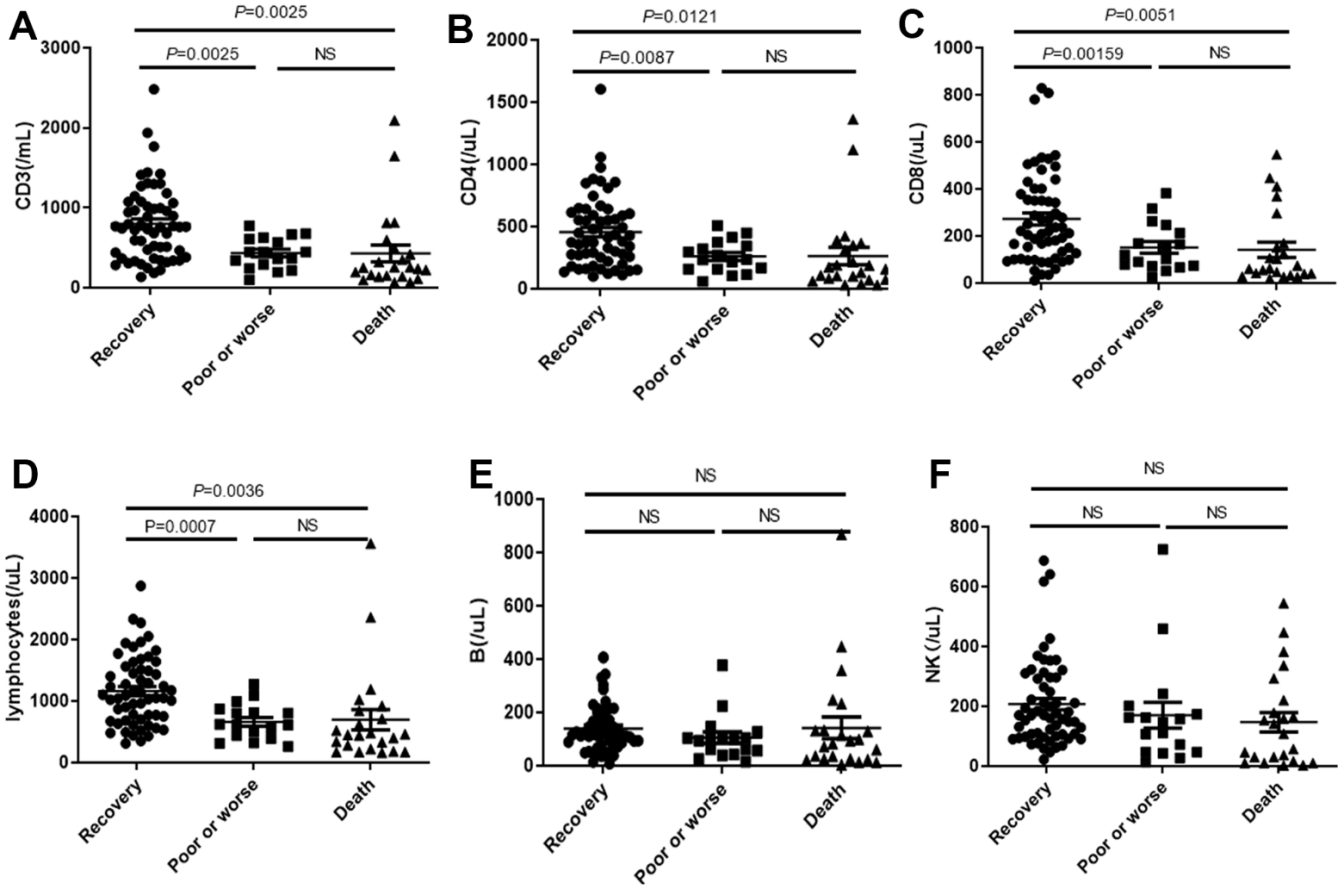
Distribution of absolute concentration of (**A**) CD3, (**B**) CD4, (**C**) CD8, (**D**) total lymphocytes, (**E**) B lymphocytes and (**F**) NK in PBMC among recovery, poor or worse, and dead groups with COVID-19. NS: not significant.

We noticed that the CD3, CD4, CD8 and total lymphocytes (Figure 6A–6D) were significantly decreased in death cases, compared with the recovery group and poor or worse group, while no differences were observed in B or NK among these groups (Figure 6E, 6F).

### Peripheral albumin, lymphocyte number and IL-6 are independent risk factors of hospitalization outcome of COVID-19

Aims to screen the potential nutritional related indexes as independent risk factors to predict the hospitalization outcome of COVID-19, we conducted one-way ANOVA test for all the indices among three groups (recovery, poor or worse and the death) following linear trend analysis using the first batch of patients with COVID-19. All the clinical parameters mentioned above were input for analysis, including age, gender, albumin, total protein, calcium, cytokines and lymphocyte counts, et al., as independent variables, and different hospitalization outcomes (death, poor or progress, recovery) were considered as dependent variables. The results were shown in Table 3, and based on the significance of slope, 13 variables were found to be associated with hospitalization outcomes, which were Ca^2+^, IFN-γ, IL-6, IL-2, IL-10, lymphocyte, total protein, albumin, age, phosphate, CD3, CD4, CD8, Mg^2+^ respectively.

**Table 3:** Results of one-way ANOVA followed linear trend analysis using death, poor or progress, and recovery as outcomes.

**Variables**	**One-way AVOVA**		**Linear trend analysis**				
	**F**	***P***	**R square**	**Slope**	**SE slope**	**Slope of 95% CI**	***P***
Ca^2+^ (mmol/L)	7.684	0.0008	0.1473	-0.05738	-0.01731	-0.02299 to -0.09177	0.0013
IFN-γ (pg/mL)	6.406	0.0028	0.1566	1.893	0.5330	0.8295 to 2.956	0.0007
IL-6 (pg/mL)	7.831	0.0009	0.1850	529.3	142.4	245.1 to 813.5	0.0004
IL-2 (pg/mL)	6.913	0.0018	0.1669	1.162	0.3126	0.5383 to 1.786	0.0004
TNF α (pg/mL)	0.6935	0.5033	0.01999	0.4849	0.4326	-0.3783 to 1.348	0.2662
IL-4 (pg/mL)	2.148	0.1244	0.05862	0.6341	0.3395	-0.04308 to 1.311	0.0660
NK (/μL)	1.411	0.2490	0.02885	-30.96	-18.52	5.810 to -67.72	0.0979
B cell (/μL)	0.5774	0.5633	0.01201	-1.958	-14.81	27.44 to -31.36	0.8951
IL-10 (pg/mL)	3.927	0.0242	0.1009	16.78	6.445	3.925 to 29.64	0.0113
IL-17a (pg/mL)	0.3431	0.7107	0.009572	0.1871	0.2348	-0.2810 to 0.6552	0.4281
Lymphocyte (/μL)	7.939	0.0006	0.1432	-256.8	-70.97	-115.9 to -397.7	0.0005
Total protein (g/L)	24.54	<0.0001	0.3430	-5.914	-0.8873	-4.152 to -7.676	<0.0001
Albumin (g/L)	13.47	<0.0001	0.2386	-31.01	-6.117	-18.85 to -43.17	<0.0001
Age (years)	3.241	0.0437	0.06649	4.352	2.079	0.2211 to 8.482	0.0392
Phosphate (mmol/L)	4.606	0.0128	0.1021	0.08427	0.03847	0.007724 to 0.1608	0.0314
CD8 (/μL)	6.343	0.0026	0.1178	-70.03	-20.84	-28.65 to -111.4	0.0011
CD4 (/μL)	5.591	0.0051	0.1063	-104.7	-34.03	-37.18 to -172.3	0.0027
CD3 (/mL)	8.119	0.0006	0.1460	-200.6	-53.62	-94.12 to -307.0	0.0003
Mg^2+^(mmol/L)	3.532	0.0334	0.07354	0.01316	0.01454	-0.01572 to 0.04204	0.3678

Further, among these 13 significant predictors of outcomes, eight had P<0.01 (one-way ANOVA) and P<0.05 (slope significance by linear trend analysis) statistical values, and these eight variables were included in an order multivariate logit regression, and three variables retained in the final model which were statistically significant independent determinants of COVID-19 hospitalization outcome (Table 4), which were albumin, lymphocyte and IL-6. Higher lymphocyte (B= -0.003, P=0.004) and Albumin (B= -0.252, P=0.036) had lower risk and better outcome of hospitality for COVID-19 patients, while IL-6 (B=0.004, P=0.045) increased the mortality and poor recovery risk, predict the unsatisfactory outcome.

**Table 4:** Results of multiple ordinal logistic model using death, poor or progress, and recovery as outcomes.

**Parameter estimates**						
**Parameter**	**B**	**Std. error**	**95% Wald confidence interval**		**Hypothesis test**	
			**Lower**	**Upper**	**Wald chi-square**	**Sig.**
Ca^2+^(mmol/L)	1.150	3.2525	-5.225	7.525	.125	.724
IFN-γ (pg/mL)	.042	.1191	-.191	.276	.127	.722
IL-6 (pg/mL)	.004	.0022	9.235E-5	.009	4.004	.045
Lymphocyte (/μL)	-.003	.0012	-.006	-.001	8.343	.004
Total protein(g/L)	.029	.0868	-.141	.199	.110	.740
Albumin (g/L)	-.252	.1200	-.487	-.017	4.401	.036
IL-2 (pg/mL)	.393	.2251	-.048	.834	3.048	.081
Age (years)	-.012	.0297	-.070	.047	.156	.693

Dependent Variable: hospitalization outcomes.

## DISCUSSION

Nutrition therapy has become the first-line treatment option for COVID-19 infection [16–18], and nutrition education programs have been reported to be helpful to change the eating habits and provide beneficial intervention during the covid-19 pandemic [19]. Nutrition-related biochemical indicators and concomitant diabetes status have become a critical basis for assessing nutritional status and formulating nutritional treatment project [20]. At present, most reports suggest that hypoproteinemia and low malnutrition status have become important indicators of the prognosis of COVID-19 infection [21, 22]. Peripheral calcium concentration was positive associated with patient prognosis significantly [20]. The correlation between phosphorus and magnesium concentration and the prognosis of COVID-19 has not yet been clarified. In this experiment, we firstly provide the evidence that the levels of periphery albumin, total protein, calcium, phosphorus and magnesium were significantly associated with the prognosis of COVID-19 infection. Nutritional biochemical indexes can comprehensively evaluate the hospitalization outcome of COVID-19 and develop nutritional treatment programs.

Cellular inflammatory cytokines release and CSS are important factors in acute respiratory distress syndrome (ARDS) and systemic multiple organ failure (SMOF) caused by COVID-19 infection. Multiple nutrients are known to inhibit cytokine release, with an improved inflammatory state of the body [23–25]. In this study, peripheral blood albumin and total protein levels were negatively associated with the cytokine levels associated with COVID-19 infection. It suggests that improving low protein malnutrition can reduce the release of inflammatory cytokines in patients infected with COVID-19, reduce the level of inflammation in the body, and then prevent the occurrence of ARDS and SMOF caused by the outbreak of inflammatory factors, and reduce the mortality rate.

The inflammatory factor profile and PBMC subsets profile associated with COVID-19 severity are different from the profile with low protein malnutrition and profile with concomitant diabetes mellitus. This study suggests that correcting low protein malnutrition is possible to reduce cytokine levels and increase immune cells, and this strategy can be more broad, fundamental, and positive. In addition, correcting low protein malnutrition can increase the proportion of Th 1 cells (CD3+, CD8+) and improve the body’s ability to clear invaded pathogens. This experiment found that COVID-19 patients with diabetes had higher IL-6 and IFN-γ expression compared with patients without diabetes, but not mononuclear lymphocytes and their subsets. That suggest that the increase of cytokines IL-6 and IFN-γ caused by COVID-19 infection leads to the deterioration of clinical outcome in hospitalized patients.

It also suggests that nutritional therapy to improve low protein malnutrition and nutritional intervention in diabetes patients are important strategy to reduce the cytokine release and hospitalization prognosis in patients with COVID-19 infection [26]. In the current study, it had been found that IL-2, IL-6, IL-12 and IFN-γ levels were significantly increased in patients with severe COVID-19, but not IL-4, IL 17 a and TNF-α levels, as compared with the major cytokines involved in severe acute respiratory syndrome are IL-1 β, IL 6, IL 12, IFN-γ, IP10 (CXCL10) and MCP 1, and IFN-γ, TNF α, IL 15 and IL 17 involved in Middle East Respiratory Syndrome. However, we found that IFN-γ and IL-6 of COVID-19 patients with diabetes were significantly higher than those of patients without diabetes. The elevation of IFN-γ and IL-6 leads to easier conversion to CSS. This is also consistent with the finding in this study that patients with diabetes had a higher in-hospital mortality rate (31% vs 18%) than those of patients without diabetes.

Analysis of PBMC subsets is an important assessment of the immune competence of organisms in disease states. We know that CD3 and CD8 are Th 1 factors, and that CD4 has the dual properties of Th 1 and Th 2. When the virus invades, the mononuclear lymphoid system is activated, CD3, CD4, CD8 is activated, when the virus invasion is dominant, CD3, CD4, CD8 is greatly consumed [27], so that CD8 and CD4 decrease with the aggravation of the disease [28]. The activated lymphocytes continue to produce cytokines. The proliferation of lymphocytes is not separated from the body’s good nutritional state and albumin level. In our study, we found a positive correlation between albumin and total protein levels and peripheral lymphocyte levels (absolute concentration of CD3, CD4, CD8, NK and B cells). In particular, albumin level was positively correlated with CD3 and CD8 percentage significantly, and total protein level was also positively correlated with CD8 percentage significantly. So higher albumin and total protein levels are broad-spectrum, fundamental, and positive for monocyte subpopulation proliferation. In particular, the increase of albumin level significantly increased the percentage of Th1 cells as CD3 and CD8.

The levels of PBMC subsets were not significantly changed between COVID-19 patients with or without diabetes. The IL-6, IL-1, IFN-γ, IL-2 and IL-10 were increased in worse and death groups, indicating that the cytokine network involved in patients with COVID-19 infection was comprehensive and complicated. PBMC subsets level of COVID-19 patients in poor and death groups is significantly lower, such as CD4+, CD3+, CD8+ lymphocyte. Severe patients with COVID-19 not only had widespread cytokines rising, but also be accompanied with excessive decline of activation of T cells (CD3, CD4, CD8). Other immune cells, such as B cells and NK cells, were not related to patient outcome, suggesting that T cell immunity and its induced pathogen clearance play a major role in the role of COVID-19 immunity.

The significant effect of albumin status and total protein on cytokine level and PBMC subsets level suggests that the effect of correcting low protein malnutrition state has the dual effect of enhancing antiviral immunity and improving immune regulation. Based on the multivariate logit regression analysis, we also confirm that albumin are independent factors which can significantly improve the hospitalization outcomes.

The current study has several limitations. The first is that the number of enrolled COVID-19 patients is not very large, therefore, further need bigger size population to confirm the present conclusion; second, we did not follow up the worse patients after their discharge of hospital. Thirdly, the studies about the micro-nutrient effects on Chinese Covid-19 patients are not enough, only calcium, magnesium, and phosphorus were investigated in the present research; therefore, the further study on more micro-nutrients was necessary, including vitamins D, C, E, zinc, selenium et al.

## CONCLUSIONS

The hospitalization outcome of COVID-19 is worse with low protein malnutrition based on cytokine profile, immune cell profile and accompanying diabetes status. This study suggests that the treatment strategy to correct hypoprotein malnutrition to reduce cytokine expression and increase immune system activation is valuable against COVID-19. Maybe the micro-nutrient supplement was also helpful for COVID-19 patients, although we did not investigate it in the current study. The rise of albumin and total protein increases the frequency of Th 1 cells (CD3, CD8), improving the body’s immunity. COVID-19 patients with diabetes increased IL-6 and IFN-γ compared those patients without diabetes, exacerbating the increase of cytokine secretion and the deterioration of clinical outcome of COVID-19. The comprehensive analysis of nutritional biochemical indicators is of great significance to evaluate the hospitalized outcome of COVID-19 and to achieve the development of nutritional treatment programs.
